# Supplementary material for: Generalized lattice Wilson–Dirac fermions in (1 + 1) dimensions for atomic quantum simulation and topological phases
Source: Sci Rep. 2018 Jul 16;8:10699. doi: 10.1038/s41598-018-29143-w (PMC6048181; doi:10.1038/s41598-018-29143-w)
Supplement: Supplementary file 1 — Supplementary Information [file 41598_2018_29143_MOESM1_ESM.pdf]

Generalized lattice Wilson–Dirac fermions in $(1+1)$ dimensions for atomic quantum simulation and topological phases

Yoshihito Kuno*[1], Ikuo Ichinose[2], and Yoshiro Takahashi[1]

[1] *Department of Physics, Graduate School of Science, Kyoto University, Kyoto 606-8502, Japan*

[2] *Department of Applied Physics, Nagoya Institute of Technology, Nagoya, 466-8555 Japan*

Supplementary Information

Λ-shaped schemes

Following Refs.[S1, S2], we shall briefly explain the basic structure of laser-assisted hopping, which we call the Λ-shaped scheme. Two ground states of neighboring sites, $|A\rangle$ and $|B\rangle$ and one excited state, $|E\rangle$, which is energetically higher than the two ground states, are used. The three level Hamiltonian H_{ABE} is given by [S3]

$$H_{\mathrm{ABE}} = \sum_{l=A,B,E} \epsilon_l |l\rangle\langle l| + \frac{\Omega_{AE}}{2}|e\rangle\langle g_j| + \frac{\Omega_{BE}}{2}|e\rangle\langle g_{j+1}| + \text{h.c.}, \tag{S1}$$

where ϵ_l is the energy for each state. The two Raman lasers have the wave-vectors $\mathbf{k}_A$ and $\mathbf{k}_B$. Here, we assume that the diagonal terms of H_{ABE} are sufficiently larger than the off-diagonal terms and that the detuning is much smaller than $|\Omega_{AE}|$ and $|\Omega_{BE}|$. Then, we use second-order perturbation theory with the rotating wave approximation (RWA) [S1, S2]. The effective transitions between the two adjacent ground states are given as

$$H_{\mathrm{eff}}^{(g)} = -\frac{1}{4\delta}\begin{bmatrix} |\Omega'_{AE}|^2 & \Omega'^{*}_{BE}\Omega'_{AE} \\ \Omega'^{*}_{AE}\Omega'_{BE} & |\Omega'_{BE}|^2 \end{bmatrix}, \tag{S2}$$

where $\Omega'_{AE(BE)}$ is the rotating frame representation. The diagonal terms are just the effective energy shifts. If $|\Omega'_{AE}| = |\Omega'_{BE}|$, they are negligible, as they just gives a uniform energy shift for each lattice site.

References

S1. Fox, M. *Quantum Optics - An Introduction* (Oxford University Press, 2006).

S2. Keilmann, T., Lanzmich, S., McCulloch, I., & Roncaglia, M. Statistically induced phase transitions and anyons in 1D optical lattices. *Nat. Commun.* **2**, 361 (2011).

S3. We assume that the other states of an atom are energetically separated from the three states by a large amount. Thus, the effective Hamiltonian becomes a simple form that includes only the three states.
